# The moderating role of sexual identity centrality in the association between gay community stress and social anxiety among sexual minority men

**DOI:** 10.1016/j.jad.2025.119755

**Published:** 2025-06-21

**Authors:** Zachary A. Soulliard, Anthony J. Maiolatesi, Robert B. Manning, Katie Wang, John E. Pachankis

**Affiliations:** a Department of Psychology, Miami University, 90 N Patterson Ave, Oxford, OH 45056, USA; b Department of Social and Behavioral Sciences, Yale School of Public Health, 60 College St, New Haven, CT 06510, USA

**Keywords:** Sexual minorities, Intraminority stress, Sexual identity centrality, Minority stress, Discrimination, Social anxiety disorder

## Abstract

Gay community stress has been linked to social anxiety among sexual minority men; however, the moderating role of identity-related processes (e.g., sexual identity centrality) has yet to be examined in this association. The present study investigated the association between gay community stress and two measures of social anxiety (i.e., self-reported social anxiety symptoms and interviewer-based diagnostic assessment of social anxiety disorder), as well as the moderating role of sexual identity centrality. Data come from the baseline assessment of a randomized controlled trial testing an LGBTQ-affirmative cognitive behavioral therapy intervention with 251 sexual minority men between the ages of 18–35. Results showed that gay community stress was significantly associated with social anxiety symptoms and social anxiety disorder diagnosis. Sexual identity centrality moderated these associations, such that the link between gay community stress and both social anxiety assessments was stronger among participants with higher levels of sexual identity centrality. These significant findings remained even after accounting for sexual orientation-based discrimination and general life stress. Future research can investigate how the interaction between gay community stress, sexual identity centrality, and social anxiety develops over time to inform interventions aimed at mitigating social distress among sexual minority men.

## 1. Introduction

Sexual minority men (i.e., gay, bisexual, queer, and other men who have sex with men) experience disproportionately high rates of social anxiety symptoms and social anxiety disorder compared to heterosexual men (Mahon et al., 2022). Social anxiety disorder, characterized by an intense fear of social interactions and negative evaluation, is one of the most prevalent psychiatric disorders associated with severe impairment across social, occupational, and interpersonal domains (American Psychiatric Association [APA], 2022; Kessler et al., 2005; Stein and Stein, 2008). Given social anxiety disorder’s high prevalence and negative impact on daily functioning, particularly among sexual minority men (Mahon et al., 2022), identifying unique factors that may heighten the risk of social anxiety disorder in this marginalized population can inform possible intervention targets.

A recent theory, intraminority gay community stress theory (hereinafter referred to as gay community stress theory; Pachankis et al., 2020), suggests that stressors stemming from within the mainstream gay community may explain the elevated rates of social anxiety among sexual minority men. Informed by interviews with sexual minority men diverse in age, race, and ethnicity, gay community stress theory posits that sexual minority men’s reliance on other men for both social and sexual rewards may lead to unique stressors in the form of status-based competition for those rewards (Pachankis et al., 2020). Such competition has been described by sexual minority men as a type of social hierarchy within the community that elevates those with privileged identities and characteristics (e.g., White, young, wealthy, masculine, muscular but lean physical appearance), while marginalizing those who do not meet these standards (Green, 2008; Hammack et al., 2022; Tran et al., 2020; Vandello et al., 2008). This status-based competition may position sexual minority men to experience status-related stress and adverse mental health outcomes. In fact, across a series of experimental and survey-based studies, perceptions of gay community stress have been directly linked to status-related concerns in interactions with other sexual minority men, which have further predicted increased psychological distress (Pachankis et al., 2020), body dissatisfaction (Soulliard et al., 2024), disordered eating (Soulliard et al., 2025), and sexual and behavioral risk-taking (Burton et al., 2020). Notably, these associations have been found even when controlling for general life stress and traditional minority stressors (e.g., sexual orientation-based discrimination; Brooks, 1981; Meyer, 2003), suggesting that stressors derived from within the mainstream gay community may affect the mental health of sexual minority men above-and-beyond other sources of stress or more general tendencies to notice or report stress (Burton et al., 2020; Pachankis et al., 2020; Soulliard et al., 2024).

The tenets of gay community stress theory are closely connected to theories regarding the etiology and maintenance of social anxiety disorder, thereby suggesting ways in which gay community stress might serve as a determinant of social anxiety among sexual minority men. Based on the cognitive model of social anxiety disorder (Clark and Wells, 1995), individuals develop social anxiety through heightened self-focused attention and negative self-perceptions based on past experiences of social rejection. These negative self-perceptions are then maintained by a fear of negative evaluation, observed in the form of cognitive and behavioral symptoms associated with social anxiety disorder. Based on this model, gay community stress represents a domain in which social rejection may occur given the competitive and evaluative nature of many social environments (e.g., bars/clubs) and relationships (e.g., friendships, romantic partnerships) in the mainstream gay community. Such environments and relationships may heighten vigilance toward potential rejection, thus reinforcing the cognitive and behavioral symptoms central to social anxiety disorder. As such, gay community stress may act as a sexual minority-specific factor contributing to increased social anxiety among sexual minority men. Indeed, in a community sample of sexual minority men, Mahon et al. (2021) found that gay community stress predicted social anxiety symptoms, even when accounting for traditional minority stressors, such as sexual orientation-based discrimination, rejection sensitivity, and identity concealment. This finding builds on prior research (e.g., Burns et al., 2012; Etkin and Wager, 2007; Feinstein et al., 2012; Hart et al., 2020) showing that traditional minority stressors—while central to understanding the disparity in social anxiety faced by sexual minority men (Mays and Cochran, 2001; Meyer et al., 2008)—do not fully account for the elevated rates of social anxiety in this population.

Although an association has been established between gay community stress and social anxiety among sexual minority men (Mahon et al., 2021), not all sexual minority men may be equally affected by stressors stemming from within the mainstream gay community. Instead, the effects of gay community stress may vary based on individual differences in how sexual minority men perceive the importance of their sexual orientation to their overall sense of self. One individual difference is sexual identity centrality, or the degree to which one’s sexual identity is deemed important to one’s overall self-concept (Ashmore et al., 2004; Hinton et al., 2022; Stryker and Serpe, 1994). To date, research on sexual identity centrality and its mental health correlates has been mixed. While greater sexual identity centrality has been linked with experiencing greater positive affirmations toward oneself as a sexual minority person (Mohr and Kendra, 2011), it has also been linked with greater perceptions of and increased sensitivity to sexual orientation-based discrimination (Hinton et al., 2022). Additionally, Maiolatesi et al. (2023) found that greater sexual identity centrality interacts with gay-related rejection sensitivity to predict both heightened social anxiety symptoms and increased likelihood of a social anxiety disorder, suggesting that placing greater importance on one’s sexual identity may exacerbate the effects of minority stressors on social anxiety.

While previous findings highlight the relevance of sexual identity centrality in the association between minority stress and social anxiety (Maiolatesi et al., 2023), its role in moderating the association between gay community stress and social anxiety among sexual minority men has yet to be examined. It is possible that sexual minority men who place greater importance on their sexual identity (i.e., those with high levels of sexual identity centrality) may be more sensitive to gay community stress than those who attach relatively less significance to their sexual identity (i.e., those with low levels of sexual identity centrality). Examining the interaction between gay community stress and sexual identity centrality in association with social anxiety can provide a more comprehensive understanding of how identity-based differences across sexual minority men may influence their risk for social anxiety.

The present study aimed to replicate prior research finding an association between gay community stress and social anxiety (Mahon et al., 2021), as well as investigate the moderating role of sexual identity centrality in this association. First, we examined the association between gay community stress and two indicators of social anxiety: symptoms of social anxiety specific to social interactions (assessed via self-report; Mattick and Clarke, 1998) and the odds of a social anxiety disorder diagnosis (assessed via interviewer-based diagnostic assessment; Sheehan et al., 1998). We hypothesized that gay community stress would be positively associated with both social anxiety symptoms and odds of a social anxiety disorder. Second, we examined the role of sexual identity centrality as a moderator in the association between gay community stress and both indicators of social anxiety. We hypothesized that perceptions of gay community stress would be associated with higher levels of social anxiety symptoms and greater likelihood of receiving a social anxiety disorder diagnosis. We also hypothesized that these associations would be stronger for sexual minority men who report higher levels of sexual identity centrality. Lastly, we hypothesized that the interactive effect of gay community stress and sexual identity centrality on social anxiety would remain significant, even after controlling for other stressors routinely found to be associated with social anxiety, namely sexual orientation-based discrimination and general life stress.

## 2. Method

### 2.1. Participants

Participants included sexual minority men enrolled in a randomized controlled trial (RCT) testing the efficacy of LGBTQ-affirmative cognitive behavioral therapy (CBT), a transdiagnostic, evidence-based treatment intended to support sexual minority men in coping with minority stress. All participants were between the ages of 18 and 35; identified as gay, bisexual, or queer; and met diagnostic criteria for at least one current depressive, anxiety, or trauma- and stressor-related disorder. Study protocol and additional inclusion and exclusion criteria for the RCT are reported in Pachankis et al. (2022). The present study used data from the baseline assessments of the RCT. We did not include three participants in the present study due to missing data on the primary variables of interest (*n* = 2) and not identifying as gay, bisexual, or queer (i.e., reported “uncertain, don’t know for sure” for their sexual orientation; *n* = 1), resulting in a final sample of 251 participants.

### 2.2. Measures

#### 2.2.1. Gay Community stress scale

The Gay Community Stress Scale (Pachankis et al., 2020) is a 20-item, self-report measure assessing perceptions of stress stemming from within the mainstream gay community. Specifically, the measure assesses sexual minority men’s perceptions of stress based on the mainstream gay community’s focus on sex (e.g., “The mainstream gay community values sex over meaningful relationships”), social status (e. g., “The mainstream gay community overly values men who are wealthy”), social competitiveness (e.g., “The mainstream gay community has a culture of competition and jealousy”), and exclusion of diversity (e.g., “The mainstream gay community is racist”). Participants indicated how personally stressed they are about the content of each of the items on a 5-point scale ranging from 1 (*not at all stressed/bothered*) to 5 (*extremely stressed/bothered*). The measure’s construct validity and internal consistency have been previously supported across diverse samples of sexual minority men (Pachankis et al., 2020). We computed an average total score, with higher scores indicating a higher level of gay community stress.

#### 2.2.2. Social interaction anxiety scale

The Social Interaction Anxiety Scale (Mattick and Clarke, 1998) is a 19-item, self-report measure assessing anxiety symptoms in interactional contexts (e.g., “I find myself worrying that I won’t know what to say in social situations”). Participants rated items on a 5-point scale ranging from 0 (*not at all*) to 4 (*extremely*). The measure’s construct validity and internal consistency have been previously supported (Mattick and Clarke, 1998), including among a sample of sexual minority adults (Lindner et al., 2013). We computed a sum total score, with higher scores indicating a higher level of social anxiety symptoms.

#### 2.2.3. Mini-international neuropsychiatric interview (MINI)

The MINI (Sheehan et al., 1998) is a structured diagnostic interview assessing psychiatric disorders in the *Diagnostic and Statistical Manual of Mental Disorders* (APA, 2022). Trained research staff verbally administered the MINI in-person to participants, including the six diagnostic questions that comprise the module assessing social anxiety disorder (e. g., “In the past month, did you have a persistent fear and significant anxiety at being watched, being the focus of attention, or of being humiliated or embarrassed or rejected?”). Participants met criteria for a current social anxiety disorder diagnosis if they responded “yes” to all six diagnostic questions and if the interviewer ruled out an organic cause for the symptoms (e.g., substance use or a non-psychiatric medical condition).

#### 2.2.4. Lesbian, gay, and bisexual identity scale (LGBIS) – Identity centrality subscale

The LGBIS – Identity Centrality subscale (Mohr and Kendra, 2011) is a 5-item, self-report measure assessing the extent to which one’s sexual identity is important to their overall self-concept (e.g., “Being an LGB person is a very important aspect of my life”). Participants rated items on a 6-point scale ranging from 1 (*disagree strongly*) to 6 (*agree strongly*). The subscale’s construct validity, internal consistency, and test-retest reliability have been previously supported in mixed-gender samples of sexual minority university students (Mohr and Kendra, 2011). We computed an average total score, with higher scores indicating a higher level of sexual identity centrality.

#### 2.2.5. Everyday discrimination scale

The Everyday Discrimination Scale (Williams et al., 1997) is a 9-item, self-report measure assessing perceptions of how often different discriminatory events have occurred in a participant’s daily life because of their sexual orientation (e.g., “In your day-to-day life how often have any of the following things happened to you because of your sexual orientation? *…* You are treated with less respect than other people”). Participants reported the frequency at which they experienced such events based on a 6-point scale ranging from 1 (*never*) to 6 (*almost every day*). The measure’s construct validity and internal consistency have been supported in previous samples of sexual minority men (Mahon et al., 2021; Pachankis et al., 2020). We computed a sum total score, with higher scores indicating greater frequency of perceived discrimination based on one’s sexual orientation.

#### 2.2.6. Perceived stress scale

The Perceived Stress Scale (Cohen et al., 1983) is a 14-item, self-report measure assessing the degree to which participants perceive various aspects of their life as uncontrollable, unpredictable, and stressful (e.g., “In the last month, how often have you been upset because of something that happened unexpectedly?”). Participants reported the frequency with which they experienced general life stress in the past month using a 5-point scale ranging from 0 (*never*) to 4 (*very often*). The measure’s construct validity and internal consistency have been supported in previous samples of sexual minority men (Pachankis et al., 2020). We computed a sum total score, with higher scores indicating a higher level of general life stress.

### 2.3. Procedure

The Human Subjects Committee at Yale University approved all study procedures. Between 2016 and 2019, the research team recruited participants in New York City and Miami, FL, both in-person (e.g., bars/clubs, LGBTQ pride events) and online (e.g., social media website, online dating apps). Participants who met study inclusion criteria provided informed consent and completed all self-report measures via Qualtrics. After completing the self-report measures, participants attended an in-person interview, in which advanced graduate students, postdoctoral fellows, and research assistants administered the MINI. All research staff received training by a licensed psychologist prior to administering the MINI. Participants completed all self-report measures and the MINI prior to randomization.

### 2.4. Data analysis

We conducted all analyses using SPSS Version 29. As a preliminary analysis, we assessed all continuous variables for normality, and considered variables normally distributed if skewness and kurtosis values were between −2 and 2 (Hair et al., 2010). Next, we reviewed the data for missing values, and examined descriptive statistics (e.g., means, standard deviations, ranges) and bivariate correlations of all study variables. We assessed associations between age and income with all study variables based on Pearson correlations, as well as for differences in sexual orientation (gay/queer vs. bisexual), race (White vs. racial minority), Hispanic or Latino identity (yes vs. no), and highest education level (less than a bachelor’s degree vs. bachelor’s degree or higher) based on phi coefficients.

To examine the main and interactive effects of gay community stress and sexual identity centrality, we used multiple linear and logistic regression to predict social anxiety symptoms and social anxiety disorder diagnosis, respectively. Prior to regression analyses, we mean-centered all continuous variables to reduce multicollinearity and aid interpretation of the parameter estimates (Aiken and West, 1991). For both models, we added gay community stress, sexual identity centrality, and relevant demographics as main effects in Step 1. Next, we added an interaction term between gay community stress and sexual identity centrality in Step 2. Lastly, we included sexual orientation-based discrimination and general life stress to the model in Step 3. Consistent with the recommendations of Aiken and West (1991), we calculated simple slopes when interactions reached statistical significance, and plotted estimated means (i.e., linear model) or probabilities (i.e., logistic model) at one standard deviation (*SD*) above and below the mean (*M*) of sexual identity centrality. Statistical significance was assessed at *p* < .05. We report unstandardized (*b*) and standardized beta (β) coefficients, standard errors (*SE*), adjusted odds ratios (aOR), 95 % confidence intervals (CI), *p*-values, adjusted *R*^*2*^, and Nagelkerke *R*^*2*^, as appropriate.

## 3. Results

### 3.1. Descriptive and preliminary analyses

The study sample (*N* = 251) was comprised of predominantly cisgender (98.8 %), gay (73.7 %) sexual minority men, for whom a majority identified as White/Non-Hispanic (33.1 %) and White/Hispanic (22.3 %). The average age was 26.52 (*SD* = 4.17), and the majority had a bachelor’s degree or higher (51.8 %) and reported an income of $29,999 or less per year (55.8 %). The average social interaction anxiety score was 34.93 (*SD* = 16.67, Range = 2–75,), and a total of 105 participants (41.8 %) met diagnostic criteria for a current social anxiety disorder diagnosis, per the MINI scoring criteria. Table 1 includes demographic characteristics of the full sample.

Missing values accounted for 0.03 % of the total data and were observed among three participants. Little’s (1988) missing completely at random test was non-significant (*p* = .33), indicating that the data were missing completely at random. All missing values were from items in the Gay Community Stress Scale and treated with pairwise deletion.

Table 2 includes descriptive statistics and correlations among the primary study variables. Older age was significantly associated with decreased social anxiety symptoms (*r* = −0.16, *p* < .05) and trended toward being associated with a decreased likelihood of being diagnosed with social anxiety disorder (*r* = −0.12, *p* = .06). Participants who identified as bisexual reported lower levels of sexual identity centrality compared to gay/queer participants (*r* = −0.18, *p* = .004). We found no other significant associations between demographic characteristics and the primary study variables. As such, we included age and sexual orientation as covariates in all regression analyses.

### 3.2. Associations among gay Community stress, sexual identity centrality, and social anxiety symptoms

**Fig. 1.**
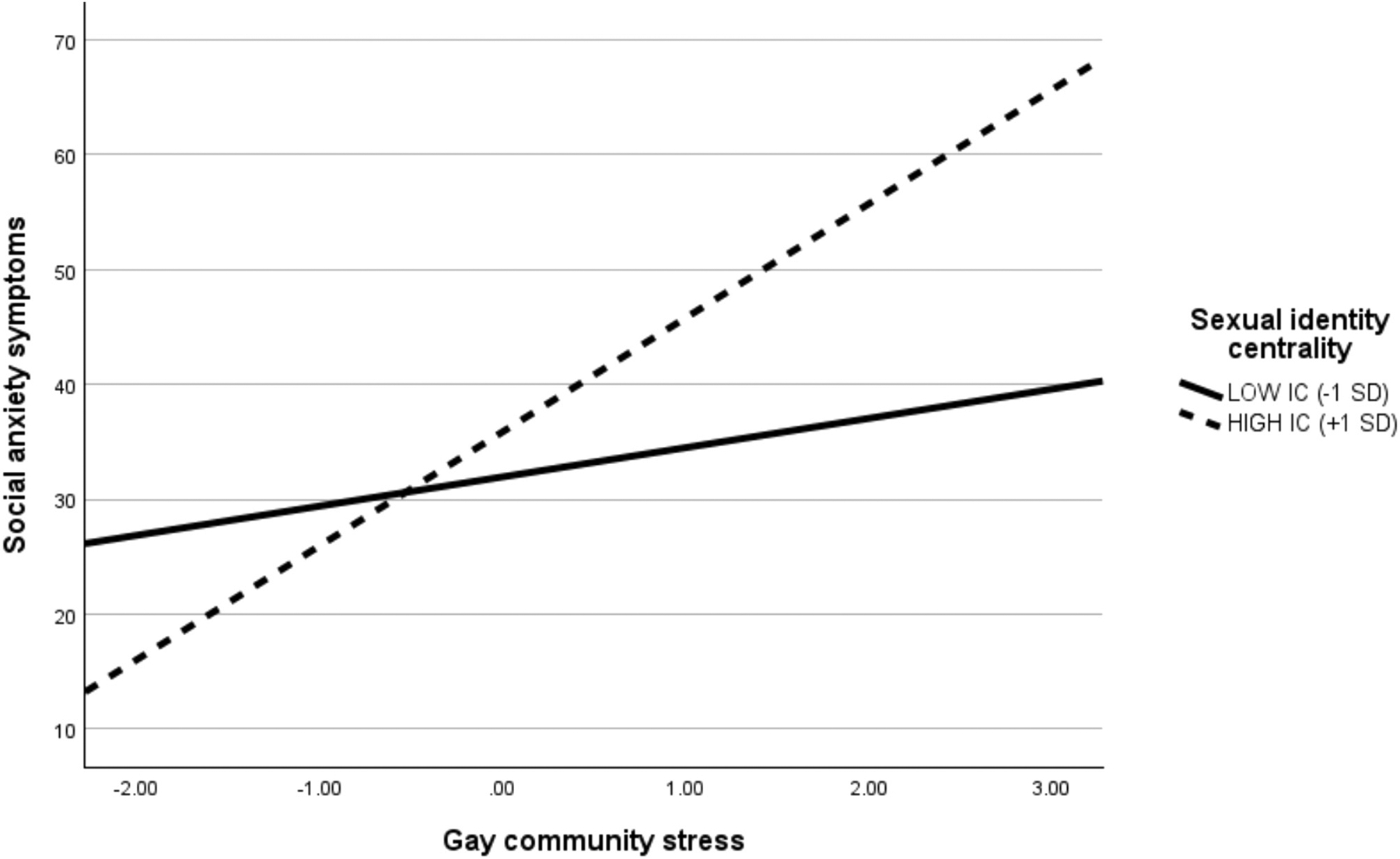
Conditional Effect of Gay Community Stress on Social Anxiety Symptoms. *Note*. IC = Identity centrality.

Table 3 includes full results from the multiple linear regression examining the associations among gay community stress, sexual identity centrality, and social anxiety symptoms. In Step 1, we included gay community stress, sexual identity centrality, age, and sexual orientation as main effects in the model, which accounted for approximately 14 % of the variance (adjusted *R*^*2*^) in social anxiety symptoms (*F*(4, 246) = 11.28, *p* < .001). Gay community stress was positively associated with social anxiety symptoms (*b* = 6.62, *SE* = 1.12, 95 % CI [4.40, 8.83], *p* < .001), and age was negatively associated with social anxiety symptoms (*b* = −0.57, *SE* = 0.24, 95 % CI [−1.04, −0.10], *p* = .02). In Step 2, we added the interaction term (i.e., gay community stress × sexual identity centrality) to the model, which was significant (*b* = 2.31, *SE* = 0.97, 95 % CI [0.39, 4.23], *p* = .02) and accounted for an additional 2 % of the variance (adjusted *R*^*2*^) in social anxiety symptoms (Δ*F* = 5.63, *p* = .02). In Step 3, we added sexual orientation-based discrimination and general life stress to the model, which accounted for an additional 11 % of the variance (adjusted *R*^*2*^) in social anxiety symptoms (Δ*F* = 18.47, *p* < .001). The fully adjusted model revealed a significant gay community stress × sexual identity centrality interaction (*b* = 1.99, *SE* = 0.91, 95 % CI [0.19, 3,78], *p* = .03), even with the addition of sexual orientation-based discrimination (*b* = 0.45, *SE* = 0.11, 95 % CI [0.23, 0.67], *p* < .001) and general life stress (*b* = 0.53, *SE* = 0.15, 95 % CI [0.23, 0.83], *p* = .001), which were both significant correlates in the model. Simple slope analyses revealed that gay community stress was positively and significantly associated with social anxiety symptoms for those with high levels (1 *SD* above the mean) of sexual identity centrality (*b* = 6.49, *SE* = 1.57, 95 % CI [3.41, 9.58], *p* < .001), but not for those with low levels (1 *SD* below the mean) of sexual identity centrality (*b* = 1.93, *SE* = 1.50, 95 % CI [−1.03, 4.89], *p* = .20; see Fig. 1).

### 3.3. Associations among gay Community stress, sexual identity centrality, and social anxiety disorder diagnosis

**Fig. 2.**
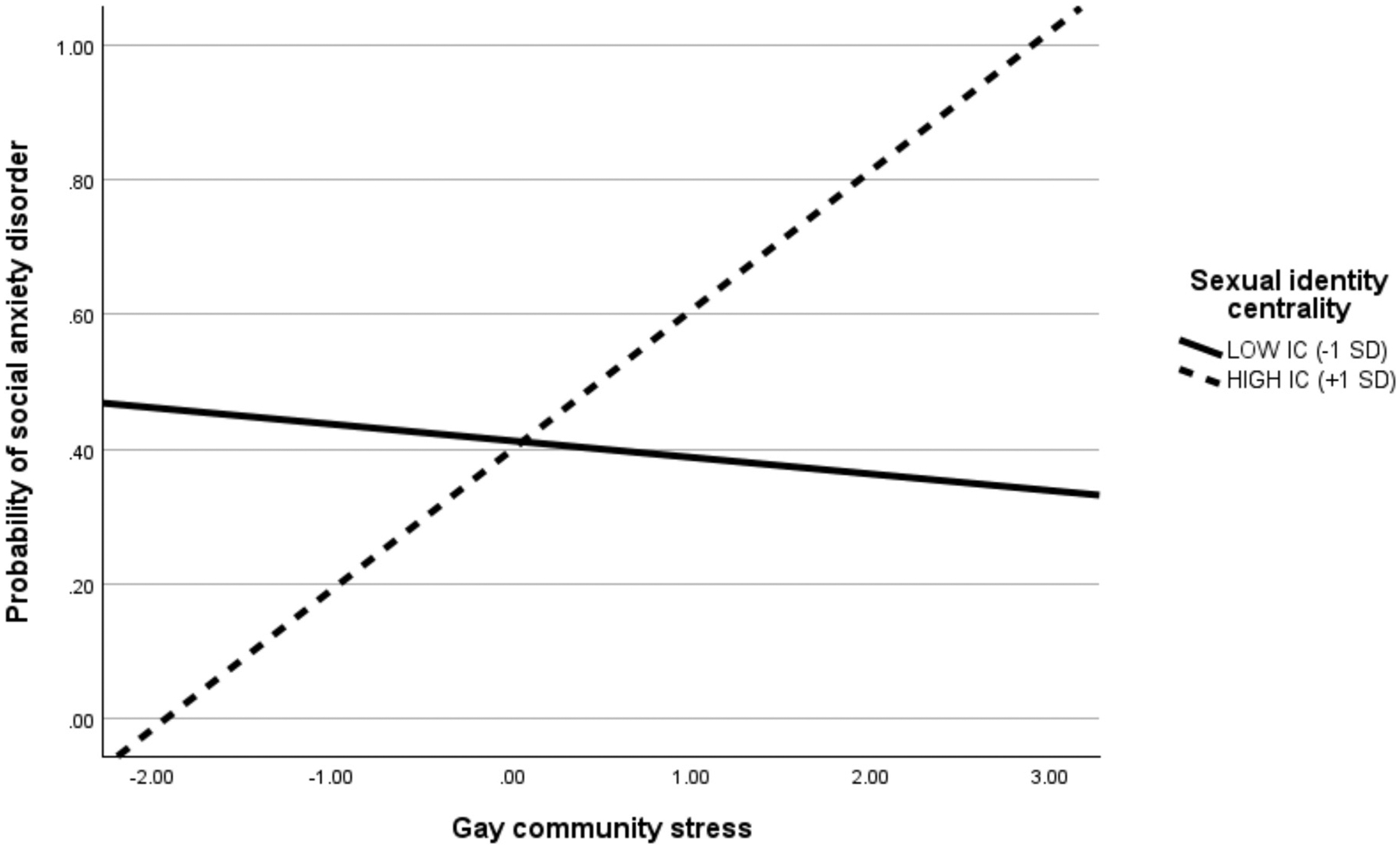
Conditional Effect of Gay Community Stress on Probability of a Social Anxiety Disorder Diagnosis. *Note*. IC = Identity centrality.

Table 3 includes full results from the logistic regression investigating the associations among gay community stress, sexual identity centrality, and a social anxiety disorder diagnosis. In Step 1, we included gay community stress, sexual identity centrality, age, and sexual orientation as main effects in the model, which accounted for approximately 7 % of the variance (Nagelkerke *R*^*2*^) in a social anxiety disorder diagnosis (χ^2^(4) = 13.87, *p* < .001). Gay community stress was significantly associated with increased odds of a social anxiety disorder diagnosis (aOR = 1.62, 95 % CI [1.19, 2.20], *p* = .002). In Step 2, we added the interaction term (i.e., gay community stress × sexual identity centrality) to the model, which was significant (aOR = 1.40, 95 % CI [1.05, 1.86], *p* = .02) and accounted for an additional 3 % of the variance (Nagelkerke *R*^*2*^) in odds of a social anxiety disorder diagnosis. A likelihood ratio test revealed that the interaction term resulted in a significantly better model fit (χ^2^(1) = 5.64, *p* = .02). Overall, the model in Step 2 accounted for 10 % of the variance (Nagelkerke *R*^*2*^) in a social anxiety disorder diagnosis (χ^2^(5) = 19.51, *p* = .002). In Step 3, we added sexual orientation-based discrimination and general life stress to the model, which resulted in a significantly improved model fit (χ^2^(2) = 15.83, *p* < .001) and accounted for an additional 8 % of the variance (Nagelkerke *R*^*2*^) in odds of a social anxiety disorder diagnosis. The fully adjusted model revealed a significant gay community stress × sexual identity centrality interaction (aOR = 1.37, 95 % CI [1.03, 1.83], *p* = .003), even with the addition of sexual orientation-based discrimination (aOR = 1.05, 95 % CI [1.01, 1.09], *p* = .005) and general life stress (aOR = 1.05, 95 % CI [1.00, 1.10], *p* = .04), which were both significant correlates in the model. Simple slope analyses revealed that gay community stress was significantly associated with increased odds of a social anxiety disorder diagnosis for those with high levels (1 *SD* above the mean) of sexual identity centrality (aOR = 1.91, 95 % CI [1.15, 3.16], *p* = .01), but not for those with low levels (1 *SD* below the mean) of sexual identity centrality (aOR = 0.92, 95 % CI [0.59, 1.44], *p* = .71; see Fig. 2).

## 4. Discussion

The present study examined the moderating role of sexual identity centrality in the association between gay community stress and social anxiety in a clinical sample of sexual minority men. Consistent with our hypotheses, we found that gay community stress was associated with increased self-reported social anxiety symptoms and likelihood of a social anxiety disorder diagnosis. We also found that sexual identity centrality moderated these associations, even after adjusting for relevant demographics, sexual orientation-based discrimination, and general life stress. Specifically, the associations between gay community stress and both indicators of social anxiety were significant for sexual minority men who reported high, but not low, levels of sexual identity centrality.

Our findings corroborate prior research demonstrating an association between gay community stress and social anxiety (Mahon et al., 2021), as well as extend the literature on both gay community stress and sexual identity centrality. The present study provides initial evidence that the influence of stressors stemming from within the mainstream gay community is conditional on the importance that sexual minority men affix to their sexual identity. Sexual minority men who ascribe greater importance to their sexual identity may be more attuned to the mainstream gay community’s focus on sex, status-based competition, and exclusion of subgroups within the community (e.g., sexual minority men of color, those in larger bodies; Tuthill, 2023). This heightened awareness of gay community stress might be particularly relevant to social anxiety given the overlap with fears of negative evaluation and feelings of self-consciousness and embarrassment, all of which are hallmark symptoms of social anxiety disorder (Langer et al., 2019). Although significant, the interaction between gay community stress and sexual identity centrality only accounted for a relatively small proportion of variance in social anxiety symptoms and odds of a social anxiety disorder diagnosis. Such a modest effect size may reflect how social anxiety is shaped by multiple individual, social, and contextual factors (Spence and Rapee, 2016). Furthermore, other traditional minority stressors not examined in the present study, such as rejection sensitivity and identity concealment, may further contribute to social anxiety, as supported by prior research (Mahon et al., 2021). Despite the small amount of variance explained, our findings suggest that sexual minority men who place greater importance on their sexual identity may be particularly hypervigilant to experiencing rejection from other sexual minority men, potentially contributing to anxiety in social situations even in the context of other determinants of social anxiety, including those unmeasured in the present study.

Importantly, our findings do not suggest that greater sexual identity centrality should be viewed as inherently negative. In fact, the main effect of sexual identity centrality was not a significant predictor of social anxiety symptoms or disorder; only in interacting with gay community stress was sexual identity centrality related to social anxiety. This finding aligns with prior identity centrality research (Ashmore et al., 2004; Stryker and Serpe, 1994), which posits that the role of identity centrality on psychological well-being is contingent on the context in which one’s identity is deemed relevant. Based on the present results, future research might explore how the interactive effect of gay community stress and sexual identity centrality on social anxiety varies in different contexts (e.g., bars/clubs vs. LGBTQ+ advocacy events; Alvarez et al., 2024). For example, in contexts where gay community stress may be particularly prominent (e.g., online dating apps; Connor, 2019; Hammack et al., 2022), placing greater importance on sexual identity may heighten one’s perception of such stressors and increase one’s vulnerability to social anxiety. Additionally, future research can explore how certain protective factors (e.g., community connectedness; Lefevor et al., 2024; Soulliard et al., 2025) might mitigate the associations between gay community stress and social anxiety.

In addition to the interaction between gay community stress and sexual identity centrality, sexual orientation-based discrimination and general life stress were positively associated with both social anxiety symptoms and likelihood of a social anxiety disorder diagnosis. These findings suggest that while gay community stress plays a role in social anxiety, traditional minority stressors and more general life stressors are also related to social anxiety for sexual minority men. In this way, gay community stressors and traditional minority stressors are both relevant determinants and likely function through separate stress pathways to inform the development and maintenance of social anxiety (Mahon et al., 2021; Pachankis et al., 2020). Consistent with prior social anxiety research (Burns et al., 2012; Etkin and Wager, 2007; Feinstein et al., 2012; Hart et al., 2020), experiences of sexual orientation-based discrimination faced by sexual minority men have been shown to exacerbate fears of rejection and negative evaluation. Such findings may be explained by the unequal treatment experienced by sexual minority men due to their sexual identity from outside of their community, perhaps leading to feelings of internalized stigma or need to conceal one’s sexual identity in non-accepting, heterosexist environments (Pachankis et al., 2021). Sexual minority men also face stressors from within their own community related to attaining a high social status compared to other sexual minority men, or feel a lack of acceptance from other sexual minority men based on one’s various identities (e.g., race or ethnicity, body size). Given that sexual minority men, like all individuals, experience social anxiety across various contexts, future research is needed to examine the specific social contexts in which anxiety might be particularly likely to occur according to one’s sexual orientation. Such research could employ micro-longitudinal designs to examine whether the associations between gay community stress and social anxiety extend beyond interactions within the mainstream gay community. For example, it is possible that gay community stress even predicts social anxiety in predominantly heterosexual environments, such as school or work settings, perhaps explained by a continued influence of past experiences of gay community stress on social anxiety symptoms, such as fear of negative evaluation or avoidance. Together, gay community stress and traditional minority stressors may contribute to symptoms of social anxiety faced by sexual minority men in interactions both within and outside of the mainstream gay community.

General life stress, another significant predictor of social anxiety in our study, may generate universal mechanisms (e.g., heightened perceptions of unpredictability, uncontrollability and stress in daily life) that play a role in the development and maintenance of social anxiety in similar ways to heterosexual individuals (Craske et al., 2009; Hofmann, 2007). Previous research has also shown that these universal mechanisms follow sexual orientation-based discrimination (Bränström et al., 2023; Hollinsaid et al., 2023; Pachankis et al., 2024). To build on our findings, future research can examine the longitudinal effects of gay community stress, sexual orientation-based discrimination, and general life stress to clarify their unique and perhaps sequential influence on social anxiety among sexual minority men across time. Furthermore, experimental studies employing gay community stress (e.g., Pachankis et al., 2020) and minority stress inductions (e.g., Fahey et al., 2024; Seager van Dyk et al., 2023) could determine whether distinct stress pathways are causal determinants of social anxiety.

Our findings have implications for psychological interventions targeting gay community stress and social anxiety in sexual minority men. LGBTQ-affirmative cognitive behavioral therapy (CBT), which has demonstrated efficacy in reducing a range of co-occurring mental health concerns, including anxiety-related symptoms, is a promising intervention for addressing gay community stress in addition to its current focus on traditional minority stressors (e.g., sexual orientation-based discrimination; Pachankis et al., 2022). Specifically, clinicians can provide psychoeducation about gay community stress with their clients, followed up by allowing space in psychotherapy for clients to share their own potential experiences of gay community stress and how such experiences have impacted their experiences of social interactions. Additionally, results suggest that clinicians might consider assessing how central a client’s sexual identity is to their self-concept to help tailor the focus of treatment. For instance, for sexual minority men who place high importance on their sexual identity, treatment might need to more strongly focus on social stressors related to this aspect of the self, compared to treatment delivered to those with a less central sexual identity. According to the present results, treatment for sexual minority men with high sexual identity centrality could fruitfully assess and address how gay community stressors might contribute to anxiety in social situations. Clinicians could also utilize techniques, such as cognitive flexibility, in LGBTQ-affirmative CBT to help their client identify and appraise thoughts related to fears of negative evaluation from other sexual minority men. Interventions or programs that focus on community-level resilience and developing supportive relationships with other sexual minority men may also prove beneficial in combatting gay community stressors and social anxiety symptoms (Jackson et al., 2022; Pachankis et al., 2023).

Despite the study’s strengths, several limitations should be noted. First, causal conclusions cannot be made given the cross-sectional nature of the data, thus precluding our ability to determine the direction of effects. It is possible that sexual minority men with social anxiety report higher levels of gay community stress, in which case social anxiety might be the cause, rather than effect, of perceptions of gay community stress. Second, because most of the study’s variables were assessed via self-report, shared method bias might have artifactually inflated the associations among the target constructs (Podsakoff et al., 2012). That said, the present study included a structured diagnostic interview of social anxiety, yielding similar results to the survey-based social anxiety symptom measure. Third, although the present study examined gay community stress along with sexual orientation-based discrimination, we did not examine other traditional minority stressors (e.g., internalized stigma, rejection sensitivity, concealment); however, prior research has found an association between gay community stress and social anxiety even when controlling for these additional minority stressors (Mahon et al., 2021). Nonetheless, future research should examine the role of sexual identity centrality in the context of a more comprehensive assessment of both gay community stress and minority stress. Fourth, our sample was comprised of sexual minority men who met diagnostic criteria for at least one current depressive, anxiety, or trauma- and stressor-related disorder. As such, the generalizability of our findings to community-based samples is uncertain. It is possible that the moderating role of sexual identity centrality is heightened or attenuated among sexual minority men not diagnosed with a mental health condition. Additionally, the current study sample was relatively homogeneous in terms of age (i.e., aged between 18 and 35), sexual identity (i.e., most identified as gay), and geographic locale (i.e., lived in one of two large, urban cities in the U.S.), which may further limit the generalizability to other populations of sexual minority men. Future research should assess the generalizability of our findings to other populations of sexual minority men underrepresented in the current sample (e.g., sexual minority men of color; bisexual, queer, and pansexual men; sexual minority men living in rural geographic locations).

In conclusion, the current study reinforces the link between gay community stress and social anxiety, while advancing the literature by identifying sexual identity centrality as a key moderator of this association. Specifically, our findings highlight that sexual minority men who experience high levels of both gay community stress and sexual identity centrality may face heightened vulnerability to social anxiety symptoms and disorder, even over-and-above their experiences of other forms of social stress and life stress more generally. These results demonstrate the importance of further exploring the interplay between gay community stress and sexual identity processes, as well as the need for future research to examine how these factors may impact social anxiety in different situational contexts. The study findings also underscore the importance of integrating a focus on gay community stress and sexual identity centrality into individual- and group-level interventions designed to address social anxiety, with the goal of mitigating the effects of intraminority stressors faced by sexual minority men.

## Figures and Tables

**Table 1:** Demographic Characteristics of the Study Sample (*N* = 251).

	*M*	*SD*
Age (Range 18–35)	26.52	4.17
	Frequency	Percent
Sexual orientation		
Gay	185	73.71
Bisexual	53	21.11
Queer	13	5.18
Gender identity^a^		
Man	248	98.80
Woman	1	0.40
Transgender man	2	0.80
Genderqueer	6	2.39
Gender non-conforming/binary	3	1.20
Two Spirit	3	1.20
Another gender identity	2	0.80
Race		
American Indian or Alaskan Native	1	0.40
Asian	10	3.98
Black/African American	43	17.13
Native Hawaiian or Other Pacific Islander	2	0.80
White	139	55.38
More than one race/Multiracial	38	15.14
Another racial identity	18	7.17
Hispanic/Latinx		
Yes	106	42.20
No	145	57.80
Highest level of education		
Some high school	2	0.80
High School Diploma or GED	19	7.57
Some College or Associates Degree	65	25.90
Currently enrolled in college	35	13.94
4-Year College Degree (BA, BS, BFA)	78	31.08
Some Graduate School	5	1.99
Currently enrolled in graduate school	18	7.17
Graduate Degree	29	11.55
Income		
<$10,000	48	19.12
$10,000 – $19,999	41	16.33
$20,000 – $29,999	51	20.32
$30,000 – $39,999	33	13.15
$40,000 – $49,999	27	10.76
$50,000 – $74,999	34	13.55
$75,000 or more	17	6.77
Social Anxiety Disorder Diagnosis		
Yes	105	41.83
No	146	58.17

a Participants could select multiple options for their gender identity.

**Table 2:** Means, Standard Deviations, and Correlations of Study Variables (N = 251).

Variables	Range	*M (SD)*	*α*	Correlation	coefficients				
				1	2	3	4	5	6
1. Gay community stress (GCSS)	1–5	2.83 (0.88)	0.95	–					
2. Social anxiety symptoms (SIAS)	2–75	34.93 (16.67)	0.94	0.36**	–				
3. Social Anxiety Disorder Diagnosis (MINI)	0, 1	–	–	0.20**	0.45**	–			
4. Sexual Identity Centrality (LGBIS-IC)	1–6	4.03 (1.15)	0.85	0.11	0.12	0.01	–		
5. Sexual Orientation-Based Discrimination (EDS)	9–54	19.84 (9.08)	0.93	0.33**	0.41**	0.28**	0.13*	–	
6. General stress (PSS)	14–51	32.37 (6.38)	0.79	0.26**	0.35**	0.23**	0.10	0.31**	–

*Note*. GCSS = Gay Community Stress Scale; SIAS = Social Interaction Anxiety Scale; MINI = Mini-International Neuropsychiatric Interview; LGBIS-IC = Lesbian, Gay, and Bisexual Identity Scale–Identity Centrality; PSS = Perceived Stress Scale; EDS = Everyday Discrimination Scale; *M* = mean; *SD* = standard deviation; α = Cronbach’s alpha.

* *p* < .05,

** *p* < .01.

**Table 3:** Regression Models Predicting Social Anxiety Symptoms (Left) and Social Anxiety Disorder Diagnosis (Right) among Sexual Minority Men (N = 251).

	Social Anxiety Symptoms (SIAS)				Social Anxiety Disorder Diagnosis (MINI)			
Predictors	*b*	*SE*	95 % CI	β	*b*	*SE*	aOR	95 % CI
Step 1								
Age	**−0.57**	0.24	−1.04, −0.10	−0.14	−0.06	0.03	0.95	0.89, 1.01
Sexual orientation	0.88	2.45	−3.95, 5.71	0.02	−0.20	0.34	0.82	0.42, 1.58
Gay community stress	**6.62**	1.12	4.40, 8.83	0.35	0.48	0.16	**1.62**	1.19, 2.20
Identity centrality	1.03	0.87	−0.68, 2.74	0.07	−0.05	0.12	0.96	0.76, 1.20
	Adjusted *R*^2^ = 0.14					Nagelkerke *R*^*2*^ = 0.07		
Step 2								
Age	**−0.61**	0.23	−1.07, −0.14	−0.15	−0.06	0.03	0.94	0.88, 1.00
Sexual orientation	1.41	2.44	−3.39, 6.22	0.03	−0.12	0.34	0.89	0.46, 1.72
Gay community stress	**6.68**	1.11	4.49, 8.88	0.35	0.52	0.16	**1.68**	1.22, 2.32
Identity centrality	1.22	0.86	−0.48, 2.92	0.08	−0.03	0.12	0.97	0.77, 1.22
Gay community stress × identity centrality	**2.31**	0.97	0.39, 4.23	0.14	0.34	0.15	**1.40**	1.05, 1.86
	Adjusted *R*^2^ = 0.16					Nagelkerke *R*^*2*^ = 0.10		
Step 3								
Age	−0.35	0.22	−0.79, 0.09	−0.09	−0.04	0.03	0.96	0.90, 1.03
Sexual orientation	0.71	2.29	−3.81, 5.22	0.02	−0.21	0.35	0.81	0.41, 1.62
Gay community stress	**4.21**	1.12	2.00, 6.42	0.22	0.28	0.18	1.32	0.94, 1.87
Identity centrality	0.65	0.81	−0.96, 2.25	0.04	−0.09	0.12	0.91	0.71, 1.16
Gay community stress × identity centrality	**1.99**	0.91	0.19, 3.78	0.12	0.32	0.15	**1.37**	1.03, 1.83
Sexual orientation-based discrimination	**0.45**	0.11	0.23, 0.67	0.25	0.05	0.02	**1.05**	1.01, 1.09
General life stress	**0.53**	0.15	0.23, 0.83	0.20	0.05	0.02	**1.05**	1.00, 1.10
	Adjusted *R*^2^ = 0.26					Nagelkerke *R*^*2*^ = 0.18		

*Note*. Bolded parameter estimates are statistically significant at *p* < .05. SIAS = Social Interaction Anxiety Scale; MINI = Mini-International Neuropsychiatric Interview.
